# TKT inhibition attenuates cardiac fibrosis in myocardial infarction through deactivating AKT signaling pathway

**DOI:** 10.1186/s12967-026-08065-6

**Published:** 2026-04-02

**Authors:** Xiaoqing Zhang, Jingwen Qin, Fenghua Lv, Xiaohong Kang, Le He

**Affiliations:** 1 Department of Cardiology, The First Affiliated Hospital of Henan Medical University, Xinxiang, Henan 453000 China; 2 Life Science Research Center, The First Affiliated Hospital of Henan Medical University, Xinxiang, Henan 453000 China; 3 Department of Cardiology, The Affiliated Hospital of Yangzhou University, No. 368, Han jiang Middle Road, Yangzhou, Jiangsu 225000 China; 4 School of Medicine, Yangzhou University, No. 136, Jiang yang Middle Road, Yangzhou, Jiangsu 225000 China

**Keywords:** Acute myocardial infarction, Cardiac fibrosis, NRCFs, NRCFs phenotypic transformation

## Abstract

**Purpose:**

Transketolase (TKT) is expressed extensively in various tissues, modulating functions of metabolism and cell apoptosis. However, the effect of TKT on cardiac fibrosis after acute myocardial infarction (AMI) has not yet been explored.

**Methods:**

Firstly, we explored the expression of TKT in patients with AMI and acute coronary syndrome (ACS) by ELISA, and investigated the predictive value of TKT on prognosis of patients with ACS by Cox regression analysis. To elucidate the role of TKT in cardiac fibrosis following AMI, we established AMI animal model by ligating the left anterior descending coronary artery (LAD) in mice. Following intervention with TKT inhibitor-Oxythiamine (OT), we further assessed the impact of TKT inhibition on myocardial fibrosis in mice using Masson, Sirius red, and immunohistochemical staining assays. Subsequently, we isolated neonatal rat cardiac fibroblasts (NRCFs), then stimulated NRCFs with TGF-β1 and TKT inhibitor to explore the mechanism of cardiac fibroblasts activation in AMI. Meanwhile, western blotting was used to analyze the expression levels of cardiac fibrotic proteins. In addition, EDU, wound healing and Transwell assays were applied to detect cell proliferation and migration function, and to explore the relationship between TKT and AKT pathway.

**Results:**

We have found for the first time that TKT was significantly elevated in AMI and ACS patients. Further confirmed that TKT not only holds significant clinical value in distinguishing AMI from unstable angina (UA) patients but also serves as a risk factor for major adverse cardiovascular events (MACE) in ACS patients. At the animal model level, we found that cardiac function was significantly improved and cardiac fibrosis alleviated in AMI mice under TKT inhibition. To further investigate the underlying mechanism, we established a cellular model of cardiac fibrosis by stimulating NRCFs with TGF-β1. Following treatment with TKT inhibitor, we observed significant suppression of both phenotypic transformation and proliferation or migration functions of NRCFs. This effect was attributable to inhibition of AKT phosphorylation. To validate TKT acts through AKT pathway, we demonstrated that TKT overexpression (OE) of NRCFs promoted phenotypic transformation, proliferation and migration functions of NRCFs in response to stimulation of TGF-β1, which could be reversed by inhibition of AKT phosphorylation.

**Conclusion:**

TKT was significantly elevated in AMI patients and AMI mice, and has preliminarily indicated the potential to be a biomarker for AMI and ACS patients. TKT inhibition reduced cardiac fibrosis in AMI mice, indicating that TKT regulated cardiac fibrosis post-AMI. TKT inhibitor suppressed phenotypic transformation, proliferation and migration functions of NRCFs, which mainly due to inhibition of AKT phosphorylation.

**Supplementary Information:**

The online version contains supplementary material available at 10.1186/s12967-026-08065-6.

## Introduction

Acute coronary syndrome (ACS) is caused by atherosclerotic plaques rupture or erosion, leading to acute occlusion of coronary artery [1, 2]. ACS consists of acute myocardial infarction (AMI) and unstable angina (UA), characterized by high risk and high mortality. Acute myocardial infarction (AMI) causes myocardial ischemic necrosis and pathological remodelling due to acute coronary artery perfusion insufficiency. Adverse cardiac remodelling primarily manifests as cardiac fibrosis characterized by excessive extracellular matrix (ECM) deposition [3]. This leads to altered myocardial structure and diminished cardiac function, progressively deteriorated into heart failure [4, 5]. The severity of cardiac fibrosis predicts adverse clinical outcomes in AMI patients [6]. The mechanism of cardiac fibrosis involves activated cardiac fibroblasts-the primary producers of ECM-secreting substantial amounts of matrix proteins that promote fibrotic process [7–10].

The activation of cardiac fibroblasts (CFs) are mediated by multiple mechanisms. For instance, increased secretion of growth factors post AMI, such as transforming growth factor-β1(TGF-β1), can activate CFs [11]. TGF-β1 induces the expression of myofibroblasts marker (such as α-SMA), thereby promoting activation of fibroblasts into myofibroblasts [12, 13]. Research indicated that cardiac fibroblasts activation has been regulated by multiple factors, for instance, the glycolytic pathway was closely associated with CFs activation [14, 15], while study has also confirmed that the pentose phosphate pathway (PPP), also played a role in AMI. TKT is a key enzyme in the non-oxidative PPP. The PPP branches off after the first step of glycolysis. Following consumption of glucose-6-phosphate (G6P) in PPP, then fructose-6-phosphate (F6P) and glyceraldehyde-3-phosphate (G3P) are yielded, ultimately returning to the glycolytic pathway [16]. The primary metabolite of the non-oxidative PPP is ribose 5-phosphate (R5P), which serves as a precursor for nucleic acid and amino acid synthesis [17]. TKT is present in all known organisms [18], and played a role in AMI. Previous study has demonstrated that TKT was highly expressed in cardiomyocytes under ischemic stress and promoted cardiomyocyte apoptosis via PARP1/AIF pathway, thereby contributed to worsened cardiac function in AMI [19]. After a comprehensive review of relevant literatures, we observed that the effect of TKT in cardiac fibrosis post AMI remains unreported, which warrants further investigation.

AKT, as a serine/threonine protein kinase, is phosphorylated and activated into p-AKT under growth factor and cytokine stimulation. This not only promotes cardiac hypertrophy and subsequently induces heart failure, and the AKT pathway is also significantly activated in myocardial fibrosis [20–24]. TKT participated in colorectal cancer cell metastasis and gastric cancer cell lactate production by promoting AKT phosphorylation has been reported [18, 25]. Given the close association between TKT and AKT phosphorylation, and the critical role of AKT activation in cardiac fibrosis, we hypothesized that TKT influences the progression of cardiac fibrosis by activating the AKT pathway.

In this study, we firstly explored the role and mechanism of TKT in myocardial fibrosis post AMI. TKT was significantly elevated in both AMI and acute coronary syndrome (ACS) patients, and has potential to be recognized as a prognostic predictor for ACS patients. We also found that TKT was primarily distributed in the cardiac fibrosis areas of AMI hearts. TKT inhibition reduced the degree of cardiac fibrosis and improved cardiac function in AMI mice. TKT inhibition also exerted cardioprotective effect on CFs, mainly via the AKT signaling pathway. Collectively, these findings suggested that TKT has potential to be a therapeutic factor for cardiac fibrosis following AMI.

## Materials and methods

### Study population

We enrolled 307 study samples including 267 ACS patients and 40 healthy controls at the Affiliated Hospital of Yangzhou University. The 267 patients with ACS further including 184 patients with AMI and 83 patients with unstable angina. Among AMI patients, 51 patients were deemed to accompany left ventricular remodelling, which was defined according to previous literature as left ventricular ejection fraction (LVEF) ≤ 50% and/or left ventricular end-diastolic diameter (LVEDD) ≥ 55 mm [26, 27]. The inclusion criteria for ACS and AMI patients were respectively based on the current guidelines [28, 29]. Patients exhibiting recent severe trauma, severe hepatic or renal dysfunction, glucocorticoid usage, autoimmune diseases or malignant tumor were excluded. Within 24 h of admission, a medical professional collected 5 ml of venous blood from each participant, with the supernatant promptly separated and preserved in sterile centrifuge tubes, then frozen in -80 °C refrigerator until experimentation. The serum concentration of TKT was detected by ELISA Kit (EK1300, Human TKT ELISA Kit, SAB, USA). The study project was approved by the Ethics Committee of Yangzhou University School of Medicine (approval Number: YXYLL-2023-133), which conforms to the principles outlined in the Helsinki Declaration, and the written informed consent was obtained from participants.

### Collection of peripheral blood mononuclear cells of AMI patients

Peripheral blood mononuclear cells (PBMCs) were collected from 5 patients diagnosed with AMI and isolated using human peripheral blood lymphocyte isolation solution. (LDS1075, Haoyang, China). Subsequently, the mRNA and protein expression levels of TKT in PBMCs were validated through real-time quantitative PCR (RT-qPCR) and Western blotting experiments.

**Follow-up of** major adverse cardiovascular events (MACE)

We followed up MACE of ACS patients including cardiac death, nonfatal myocardial infarction, readmission for unstable angina and heart failure. The median length of follow-up was 352 days, which was calculated from the date of ACS diagnosis to the occurrence of MACE or the end of follow-up. A follow-up telephone and review of medical records were performed to collect MACE of ACS patients.

### Reagents and antibodies

Oxythiamine (O4000, Sigma-Aldrich, USA). LY294002 (HY-10108, MedChemExpress, USA). Recombinant Human TGF-β1 Protein (HY-P7118, MedChemExpress, USA). TKT overexpression plasmid and empty plasmid were purchased from Gema Gene. The following primary antibodies were used: anti-TKT (1:2,000, Proteintech, 11039-1-AP), anti-α-SMA (1:2,000, Proteintech, 14395-1-AP), anti-Vimentin(1:2,000, Santa cruz, sc-6260), anti-Fibronectin (1:4,000, Proteintech, 66042-1-Ig), anti-CTGF (1:2,000, Proteintech, 25474-1-AP), anti-p-AKT (1:1,000, #4060, Cell Signaling Technology, USA), anti-AKT (1:2,000, #4691, Cell Signaling Technology, USA), anti-β-Actin (1:2,000, Santa cruz, 66009-1-Ig), anti-α-Tubulin (1:2,000, Servicebio, GB11200-100). FITC conjugated goat anti mouse/rabbit IgG (SA00003-1/2, Proteintech, China) were used for immunofluorescent analysis.

### Real-time quantitative PCR

Total RNA of PBMCs was extracted and converted to cDNA using Reverse Transcription Kit (Vazyme Biotech Co., China). RT-qPCR was performed by using SYBR Green qPCR Kit (Vazyme Biotech Co., China). Primers were provided by Tsingke Biotech Co. as follows (Table 1). Experimental data were quantified by comparative cycle threshold (CT) method.

**Table 1:** Primers used in RT-qPCR

Gene symbol	Forward (5’-3’)	Reverse (5’-3’)
Hum-TKT	TGTGTCCAGTGCAGTAGTGG	ACACTTCATACCCGCCCTAG
Hum-ACTB	TGGCACCCAGCACAATGAA	CTAAGTCATAGTCCGCCTAGAAGCA

### Isolation and culture of neonatal rat cardiac fibroblasts

Neonatal rat cardiac fibroblasts **(**NRCFs) were isolated from 1-3-day-old Wistar neonatal rats. Firstly, neonatal rats were anesthetized with 5% isoflurane and sacrificed by cervical dislocation. Hearts were digested with 0.25% Trypsin (Solarbio, China) overnight at 4℃ and the next day was replaced with 0.08% collagenase II (Worthington Biochemical, USA). Heart tissue was digested with collagenase II in 37 °C water bath until no visible cardiac tissue remained. The cell suspension was filtered through a 100 μm filter membrane, then centrifuged and the cell pellet was collected. After red blood cell lysis and centrifugation, the cell pellet was resuspended in DMEM with 10% fetal bovine serum (Gibco, USA), and aliquoted into several 10 cm culture dishes. After plating for 2 h, NRCFs attached to plates and nonadherent cells were removed. NRCFs at the second or third passages were used for experiments.

### TKT overexpression plasmid transfection

NRCFs in the logarithmic growth phase were transfected. Briefly, TKT overexpression (OE) and empty vector plasmids were mixed with P3000 transfection reagent (Invitrogen, USA), followed by incubation at room temperature for 5 min. Subsequently, Lipofectamine 3000 was added, the mixture was incubated at room temperature for 15 min. During the incubation period, the complete medium in the 6-well plate was replaced with serum-free DMEM. After incubation completed, the serum-free DMEM containing the transfection reagent-plasmid complex was uniformly added to each well. Then cells were cultured at 37 °C, and 6 h after transfection, the medium containing the transfection reagent was removed and replaced with complete medium. Thereafter, NRCFs were cultured for 72 h and then harvested for subsequent experiments.

### Western blotting

Protein was extracted using RIPA lysis buffer (C1053, Pulilai, China). Equal amounts of each protein sample were then separated via SDS-PAGE electrophoresis, followed by transferring onto a polyvinylidene difluoride membrane. After 2 h of 5% skimmed milk blocking (P0216, Beyotime, China), membranes were incubated with primary antibodies (see Reagents and antibodies part), which were diluted using western blotting specialized antibody diluent overnight at 4 °C. The next day, the membranes were incubated with the enzyme-labeled secondary antibody at room temperature for 2 h. Protein bands were quantified using the ImageJ software.

### EDU assay

EDU assay was used to evaluate cell proliferation status. NRCFs were incubated with 50 mM EDU (C0078S, Beyotime, China) for 2 h. Nuclei were stained with Hoechst33342 for 30 min. The percentage of proliferating cells marked with EDU exhibited red fluorescence was calculated. The number of EDU labeled cells was counted in 3 randomly selected fields under a microscope, and the mean values were used for statistical analysis.

### CCK8 assay

The processed cells were seeded into a 96-well cell culture plate with a final volume of 100 µL. The cells were then treated with TKT inhibitor-OT at concentrations of 10, 20, 40, 60, and 80 µM refer to previous study [30–32]. 10 µL of CCK-8 staining solution (Beyotime, China) was added to the 96-well plate and incubated for 1–2 h, then the absorbance was measured at 450 nm (Bio-Rad, USA). Finally, the standard curve and statistical analysis was completed based on the absorbance values.

### Wound healing

After NRCFs reaching the logarithmic growth period, cells were digested with 0.25% trypsin and plated into 6-well plates. Then NRCFs were treated with TKT inhibitor. After reaching 90% confluency, a sterile pipette tip was used to produce a straight line, followed by PBS to wash away the floating cells. NRCFs were cultured with serum-free medium containing TGF-β1 at concentrations of 0 and 10 ng/ml, respectively. Images were both captured at 0 h and 24 h at the same location (Nikon, Japan). The cell migration rate was calculated as: Migration rate (%) = (Migration area - Initial area) / Initial area × 100%.

### Transwell assay

For the transwell test, NRCFs were plated into the upper chamber of a transwell plate (8 μm pore size, Corning, USA) in serum-free medium with TKT inhibitor and/or TGF-β1incubation, and the lower chamber was filled with complete medium containing 10% FBS. After 24 h of incubation, NRCFs were fixed with 4% paraformaldehyde (P0099, Beyotime, China), stained with 0.1% crystal violet solution (G1064, Solarbio, China) and photographed. The number of migrated cells was counted in 3 randomly selected fields under a microscope, and the mean values were used for statistical analysis.

### AMI animal model and TKT inhibition

Male C57BL/6J mice (20–25 g, 8–10 weeks) and 1-3-day-old Wistar neonatal rats were purchased from Yangzhou University. C57BL/6J mice were raised in specific pathogen free (SPF) environment and the neonatal rats were used for isolation of NRCFs. The mice were randomly divided into 3 groups (*n* = 12 in each group): Sham group, AMI + PBS group, and AMI + OT (OT is a TKT inhibitor, Sigma, USA**)** group. AMI + OT group were treated with OT at a dose of 200 mg/kg/day (intraperitoneal (i.p.)) starting from 3 days and 1 day before left anterior descending coronary artery (LAD) ligation operation, and 1 day after operation restarted, 3–4 times per week over the next 13 days [32]. The mice of three groups were sacrificed 14 days after operation. Cardiac tissue of mice were collected for protein assay and histological experiment.

Mice were initially anesthetized with isoflurane. Chest hair was removed, and the surgical area was disinfected with 75% ethanol. The mice were fixed in the supine position, and endotracheal intubation was prepared when the hindlimb reflex was diminished. The incisors were fixed to hyperextend the head, the mouth was opened to expose the glottis, and an endotracheal tube was inserted and connected to a rodent ventilator. Successful intubation was confirmed by synchronous chest movement with the ventilator frequency. The chest skin was incised, and thoracic wall muscles were bluntly separated layer by layer. The intercostal space with the most obvious apical impulse was spread and fixed with hemostats to fully expose the heart, the LAD was ligated 1–2 mm below the left atrial appendage. Successful ligation was indicated by immediate myocardial blanching and weakened cardiac contraction. The thoracic cavity was closed, and the skin was sutured with 4 − 0 sutures. The endotracheal tube was removed after recovery of spontaneous respiration, and mice were placed on a thermostatic heating pad until awakening before being returned to cages. The mice were observed daily postoperatively, and prompt treatment was administered if abnormalities occurred.

### Echocardiography examination

Following successful ligation of the LAD in mice, animals were maintained for 14 days. Echocardiography was performed one day prior to euthanasia. Mice were anesthetized with isoflurane and secured on the platform. The Vevo 3100 echocardiography system was used to measure parameters including: left ventricular systolic inner diameter (LVIDs), left ventricular diastolic inner diameter (LVIDd), left ventricular systolic volume (LV Vols), left ventricular diastolic volume (LV Vold), left ventricular ejection fraction (LVEF), and left ventricular fractional shortening (LVFS). Repeating the measurement for three times and taking the average.

### Immunohistochemical staining (IHC)

Mouse heart tissues were harvested, washed with PBS, and fixed in 4% paraformaldehyde for 24 h, followed by automatic dehydration, paraffin embedding, and serial sectioning. The prepared paraffin slices were first deparaffinized and rehydrated, and then proceeded with 1x sodium citrate retrieval solution to complete antigen retrieval. Endogenous peroxidase activity was blocked according to the protocol of Two Step IHC Kit (PV-9000, ZDGB-BIO, Co., China). Slices were blocked with 10% normal goat serum at 37 °C for 30 min to block nonspecific bindings. Then primary antibodies (anti-α-SMA (1:200), anti-VIM (1:200)) were used and incubated overnight at 4 °C. After removing primary antibody, reaction enhancement solution was applied and incubated at 37 °C for 20 min. Then, enhanced enzyme-labeled goat-anti-mouse/rabbit IgG polymer was added, and incubated at 37 °C for 20 min. Following DAB staining, hydrochloric acid-alcohol differentiation, dehydrating and clearing, and sealing, the slices were observed under a microscope and photographed. Three randomly selected fields from each slide were measured, and the mean values were used for statistical analysis.

### Sirius red staining

Paraffin sections were heated at 60 °C for 30 min. After deparaffinization and rehydration, Sirius red staining solution (G1472, Solarbio, China) was added to the tissue and incubated at room temperature for 10 min. The slices were then stained with Mayer’s hematoxylin staining solution, differentiated in hydrochloric acid-alcohol, dehydrated, cleared, and sealed, and examined under a microscope (Nikon, Japan). Three randomly selected fields from each slice were measured, and the mean values were used for statistical analysis.

### Masson staining

To assess the degree of cardiac fibrosis, Masson staining was conducted, Mordant solution, Celestin blue staining solution, Mayer’s hematoxylin staining solution, Acid differentiation solution, Ponceau-fuchsin solution, Phosphomolybdic acid solution, Aniline blue solution, and Weak acid solution were added sequentially according to the protocol of Modified Masson’s Trichrome Stain Kit (G1346, Solarbio, China). The area-stained blue was the fibrotic area. The ratio of fibrotic area to the whole heart was calculated, which was considered as the percentage of infarct size.

### Statistical analysis

Statistical analysis was conducted using SPSS 26.0 or GraphPad Prism 8.0. Normality was evaluated by Shapiro-Wilk test (*P* > 0.05) and equal variance by F test (*P* > 0.05). For measures that conformed to normal distribution, two independent sample t-test was used for two-group comparison, while one-way ANOVA with Tukey post hoc test for was used for multiple groups. For non-normally distributed measurements, the Mann-Whitney U test was used to compare two-group and Kruskal-Wallis with Dunnett post hoc test for multiple groups. Categorical variables were expressed as rates (%) and comparisons between groups were made using the χ² test. The Kaplan-Meier method was used to plot the survival curve, and Log-rank test was used to compare the difference in cumulative survival rates. Cox proportional hazards regression analysis was applied to explore risk factors of MACE in ACS patients. *P* < 0.05 was considered statistically significant.

## Results

### Increased TKT in patients with AMI correlated with cardiac remodelling, and predicted MACE of ACS patients

**Fig. 1:**
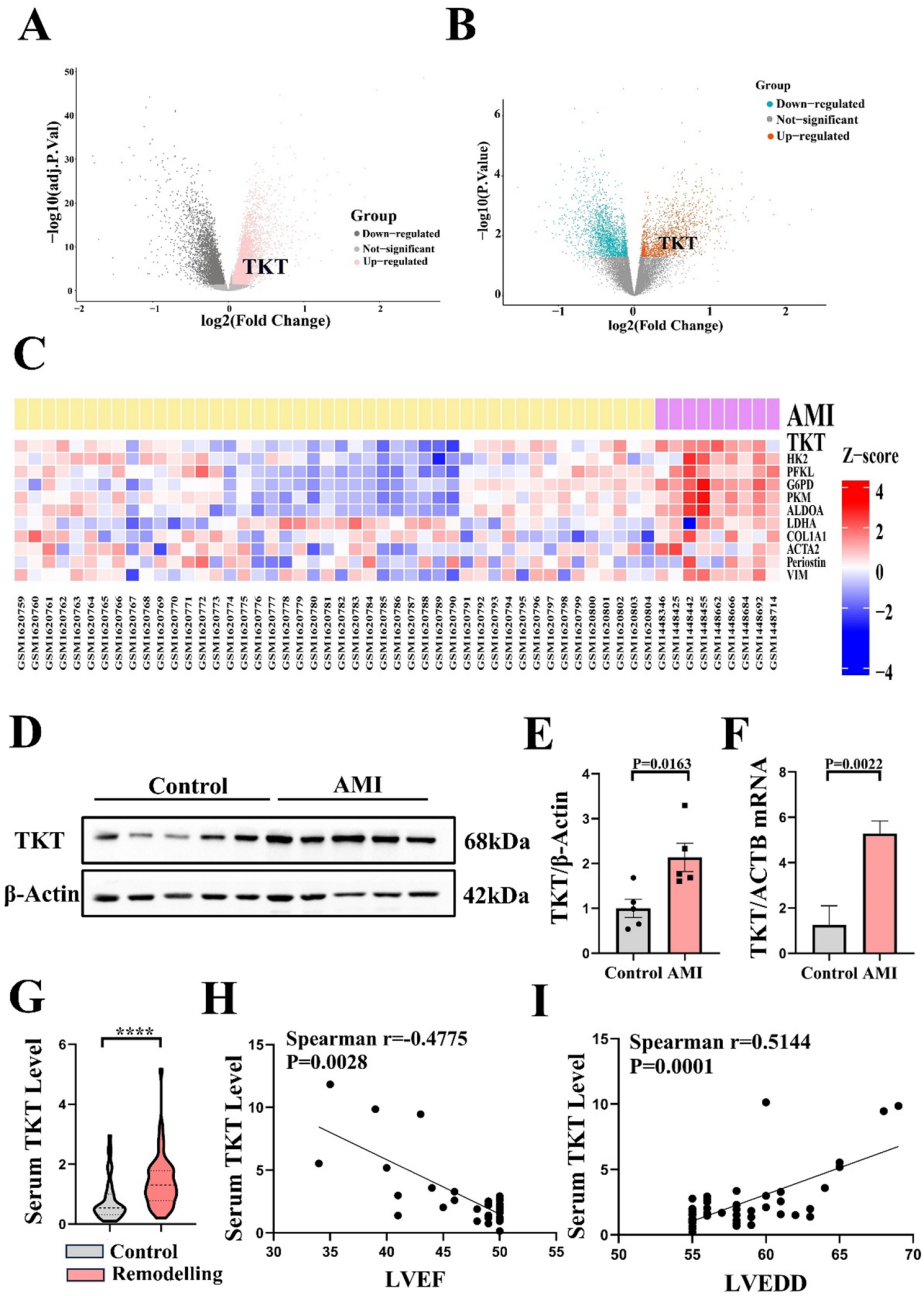
Bioinformatic analysis and experimental validation of TKT level in AMI patients. **A**: Analysis of GEO RNA-seq data (GSE57345) for TKT expression level in human cardiac tissue from patients with heart failure. **B**: Bioinformatic analysis of GEO RNA-seq data (GSE61145) for TKT level in serum of AMI patients. **C**: Heatmap generated from GSE59867 for TKT level in PBMCs of AMI patients, and the pink represents AMI patients, the yellow represents stable coronary artery disease patients. **D-E**: Western blotting of TKT level in PBMCs of AMI patients (*n* = 5). **F**: RT-qPCR of TKT mRNA level in PBMCs of AMI patients (*n* = 3). **G**: ELISA result of TKT level in patients with AMI and left ventricular remodelling (*n* = 51). **H**: Correlative analysis between TKT and LVEF in AMI with left ventricular remodelling patients. **I**: Correlative analysis between TKT and LVEDD in AMI with left ventricular remodelling patients. Two-tailed Unpaired t test (**E-F**) and Mann-Whitney U test (**G**) was used to test the difference. **** *P* < 0.0001 vs. Control group. AMI indicates acute myocardial infarction; LVEF, Left ventricular ejection fraction; LVEDD, Left ventricular end-diastolic diameter

We firstly downloaded datasets from the GEO database: GSE57345 for cardiac tissue of heart failure patients and GSE61145 for serum of AMI patients. Further analysis of TKT was significantly elevated in both cardiac tissue of heart failure patients and serum of AMI patients (Fig. 1A and B). We also analyzed the PBMCs dataset GSE59867 for AMI patients, and found that TKT was markedly elevated (Fig. 1C). Experiments using PBMCs of AMI patients further confirmed elevated TKT expression at both mRNA and protein level (Fig. 1D-F). We further collected serum of patients with AMI and left ventricular remodelling, ELISA result revealed that TKT was significantly elevated in patients with AMI and left ventricular remodelling (Fig. 1G). We also found a significant negative correlation between TKT level and LVEF, and a significant positive correlation between TKT level and LVEDD in AMI with left ventricular remodelling patients (Fig. 1H and I), suggesting that TKT may be implicated in the progression of cardiac remodelling in AMI.

TKT was significantly upregulated in the GSE61145 dataset of ACS patients, and our ELISA result also demonstrated a significant increase in TKT level of ACS patients (Figure S1A and B). We then subdividing ACS patients into unstable angina and AMI patients, and observed that TKT was significantly higher in AMI patients compared with UA patients (Figure S1C). We conducted logistic regression analysis of AMI and UA patients, then used statistically significant clinical variables (including TKT) which identified through binary logistic regression analysis (Table S1 and 2), and a clinical prediction model including TKT was constructed (Figure S2A). The clinical model demonstrated excellent discriminatory ability between AMI and UA patients (Figure S2B-E). We further followed up MACE of ACS patients, result clarified that increased TKT level may be a risk factor of MACE in patients with ACS (Table S3). Based on the median expression level of TKT, the cohort was divided into two groups: higher TKT (≥ 2.289ng/mL) and lower TKT (< 2.289ng/mL) group. Kaplan-Meier survival curve demonstrated survival rates was reduced in higher TKT group. We also constructed and evaluated prognostic prediction model using statistically significant clinical variables (including TKT) (Figure S3), which identified through Cox proportional hazards regression analysis. The results indicated that TKT may be implicated in predicting MACE of ACS patients, which need to be further validated in large clinical cohorts.

### Heart TKT upregulation and mainly expressed in the fibrotic zone of AMI hearts

**Fig. 2:**
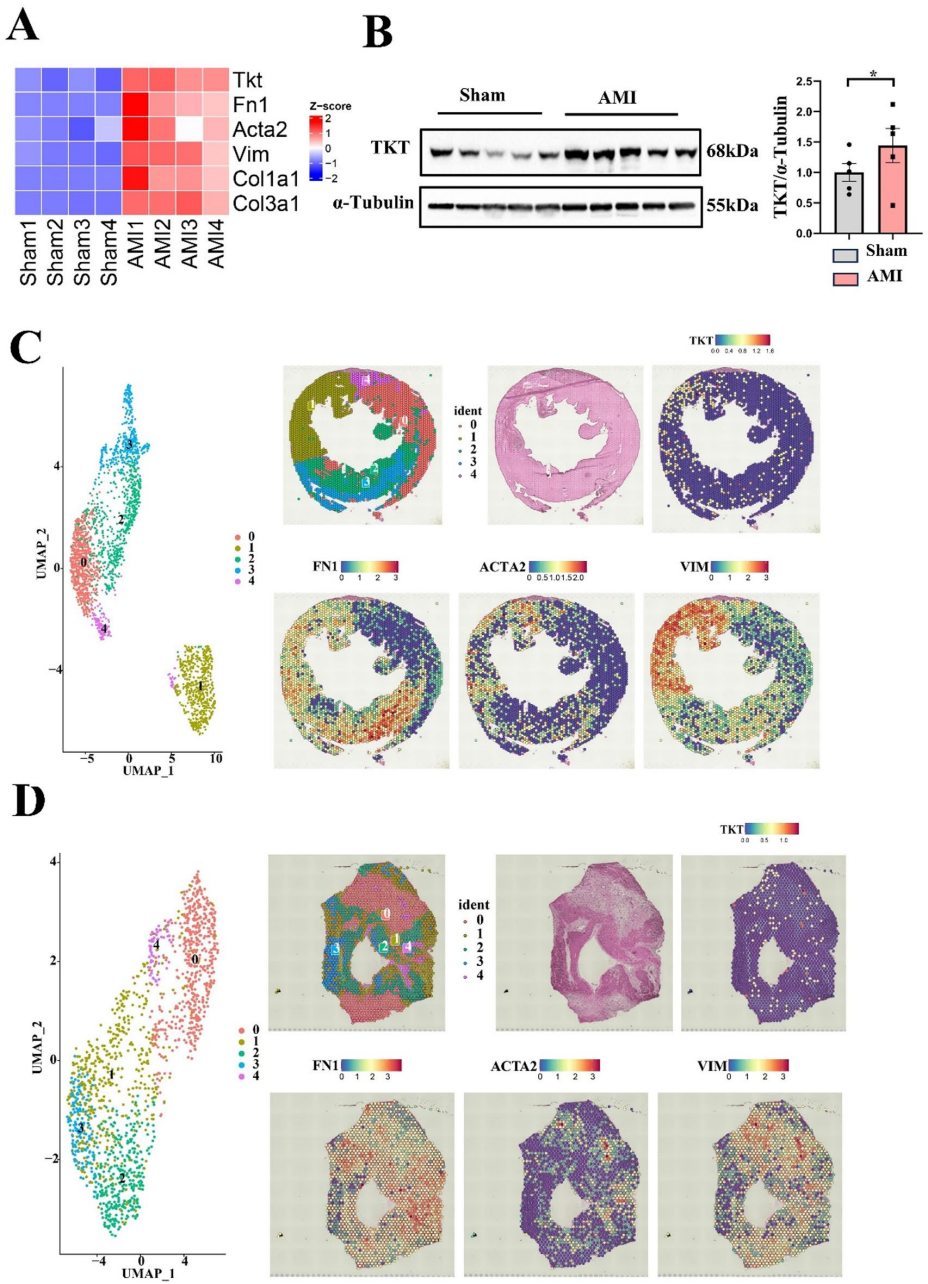
TKT was elevated in AMI mice, and TKT was primarily distributed on cardiac fibrosis areas post-AMI. **A**: Analysis of GEO RNA-seq data (GSE151834) for TKT expression level in AMI mice. **B**: Western blotting of TKT level in AMI mice (*n* = 5). **C**: Single-cell spatial transcriptomic analysis of TKT and fibrotic proteins in AMI mice. **D**: Single-cell spatial transcriptomic analysis of TKT and fibrotic proteins in AMI patients. Data were expressed as mean ± SEM. Two-tailed Unpaired t test was used to test the difference (**B**), *P < 0.05 vs. Sham. AMI indicates acute myocardial infarction; FN1, Fibronectin; Vim, Vimentin

We performed differential gene analysis of AMI mice dataset, and found that TKT was significantly elevated (Fig. 2A). To explore the expression of TKT in AMI mice, AMI animal model was successfully constructed by ligating LAD in mice (Figure S4A-C), and western blotting result demonstrated that TKT was significantly elevated in AMI mice (Fig. 2B). The single-cell spatial transcriptomic dataset GSE214611 of AMI patient and AMI mice revealed that TKT expression zone on hematoxylin-eosin (HE) staining sections largely overlapped with regions with elevated expression of fibrotic proteins including Fibronectin (FN1), α-SMA (ACTA2), and Vimentin (VIM) (Fig. 2C and D), which suggested that TKT was closely associated with cardiac fibrosis in AMI.

### TKT inhibitor OT improved cardiac function in AMI mice

**Fig. 3:**
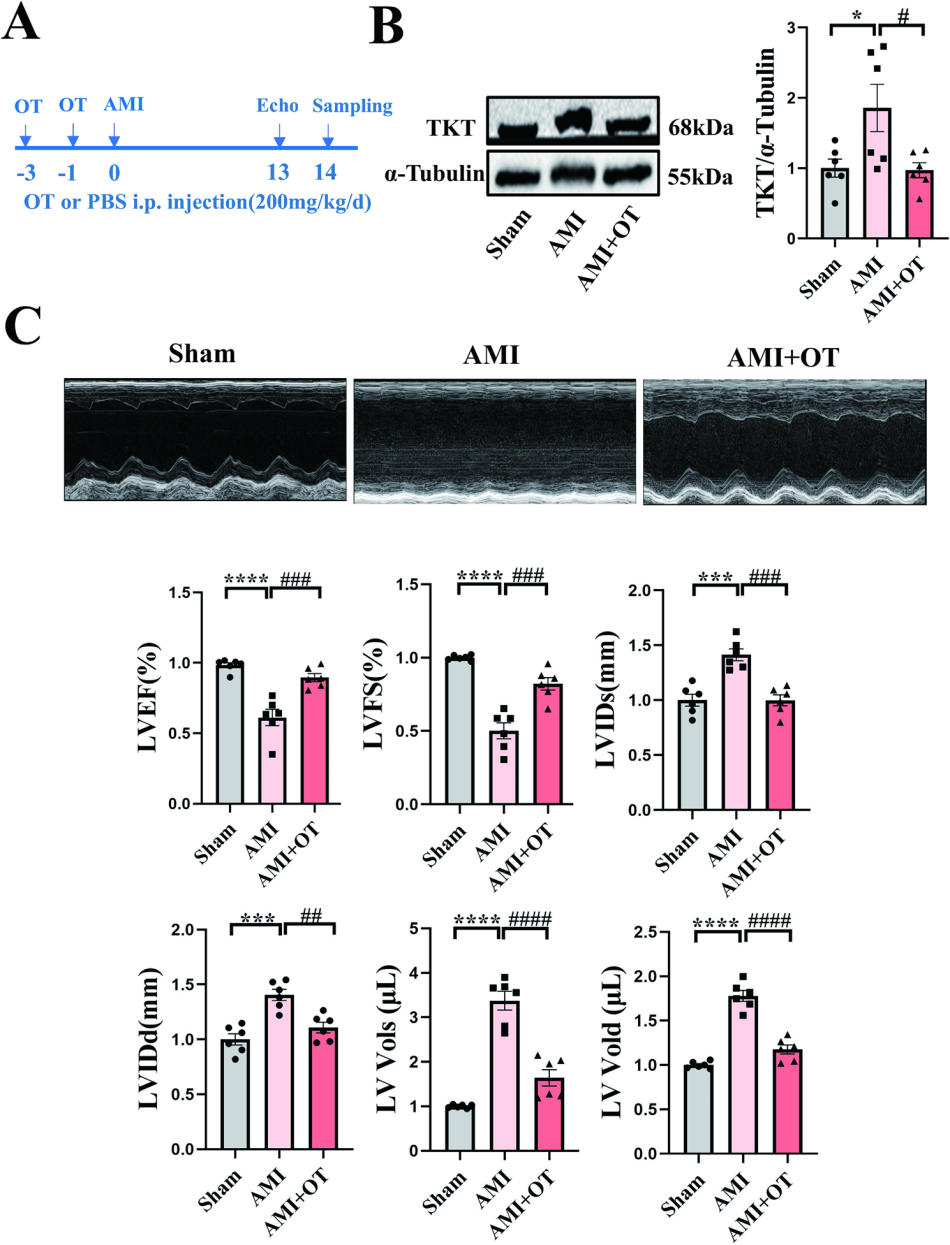
TKT inhibitor ameliorated cardiac function deterioration in AMI mice. **A**: Time axis of AMI mice. OT or PBS was administrated to AMI mice from 3 days and 1 day before AMI operation until sampling (14 days after operation, 3–4 times per week). Echocardiography was performed 1 day before AMI mice were sacrificed (13 days after operation). **B**: Western blotting was performed to verify TKT inhibitor playing a role (*n* = 6). **C**: Echocardiographic assessment of cardiac function in AMI mice (*n* = 6). Data were expressed as mean ± SEM. One-way ANOVA test was used to test the difference (**B-C**). * *P* < 0.05,*** *P* < 0.001, **** *P* < 0.0001 vs. Sham. # *P* < 0.05, ## *P* < 0.01, ### *p* < 0.001, #### *P* < 0.0001 vs. AMI. AMI indicates acute myocardial infarction; Echo, Echocardiography; LVEF, Left ventricular ejection fraction; LVFS, Left ventricular fractional shortening; LVIDs, Left ventricular systolic inner diameter; LVIDd, Left ventricular diastolic inner diameter; LV Vols, Left ventricular systolic volume; LV Vold, Left ventricular diastolic volume; OT, Oxythiamine

In vivo experiments were performed to validate the effect of TKT inhibitor Oxythiamine (OT). OT was administrated to AMI mice from 3 days and 1 day before operation until sampling (intraperitoneal (i.p.) injection, 200 mg/kg/day, 3–4 times per week), and we have demonstrated the effectiveness of OT (Fig. 3A and B). AMI mice were sacrificed at the same time after OT treatment (14 days after operation). Cardiac echocardiography revealed that compared with sham group, AMI mice exhibited significantly reduced LVEF and LVFS, accompanied by increased LV Vol and LVID (Fig. 3C). TKT inhibitor OT significantly improved cardiac function in AMI mice, manifested as elevated LVEF and LVFS along with reduced LV Vol and LVID (Fig. 3C). These findings indicated that OT treatment significantly mitigated cardiac dysfunction in AMI mice.

### TKT inhibitor improved cardiac fibrosis in AMI mice

**Fig. 4:**
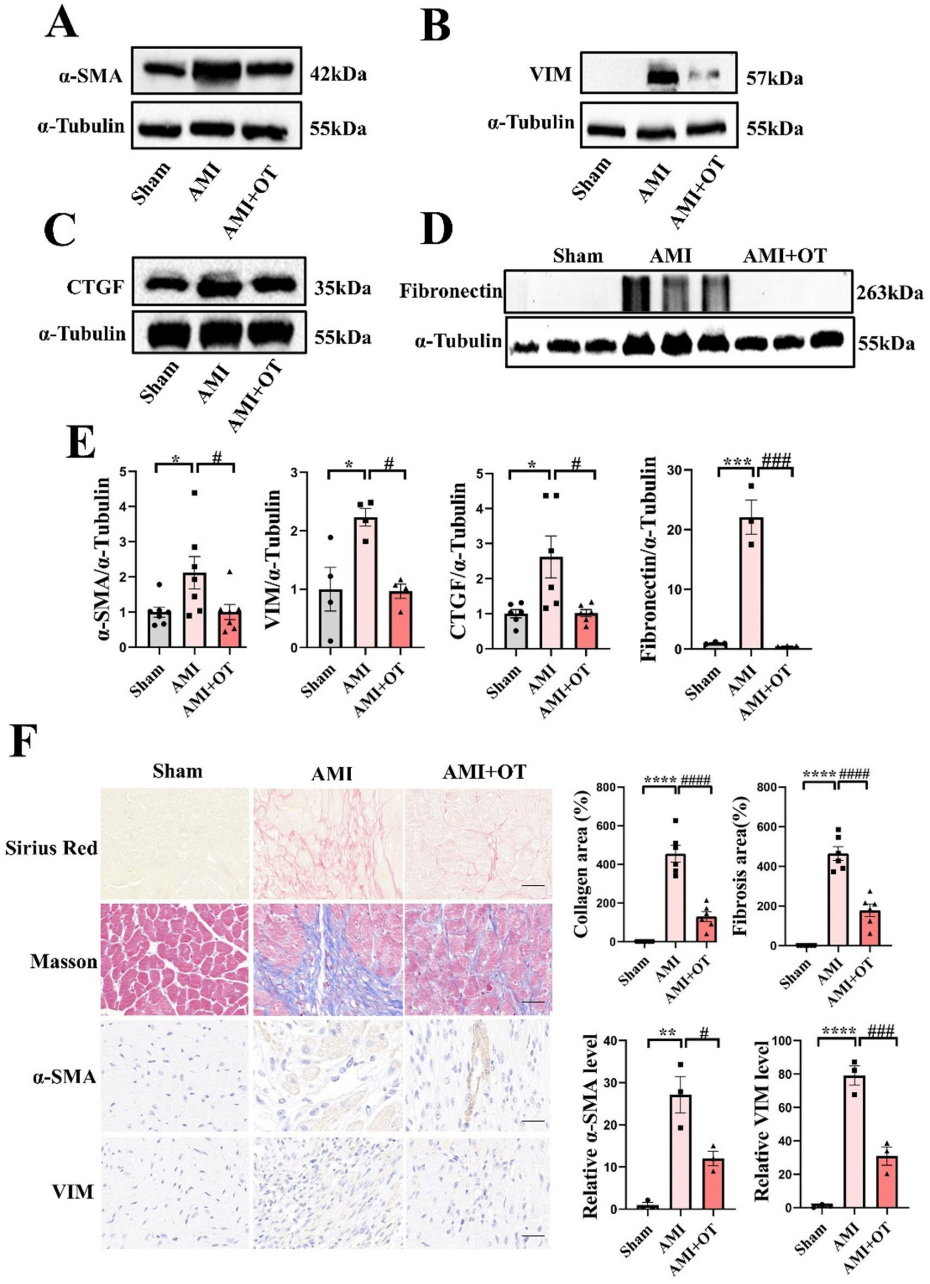
TKT inhibitor OT improved cardiac fibrosis in AMI mice. **A-E**: Western blotting of Fibronectin, α-SMA, VIM, and CTGF under OT treatment. F: Sirius red, Masson and IHC staining images under OT treatment. *n* = 3–6. Data were expressed as mean ± SEM. One-way ANOVA test was used to test the difference (E, F). * *P* < 0.05, ** *P* < 0.01, *** *P* < 0.001, **** *P* < 0.0001 vs. Sham. # *P* < 0.05, ### *P* < 0.001, #### *P* < 0.0001 vs. AMI. Scale bar: 50 μm. AMI indicates acute myocardial infarction; α-SMA, α-smooth muscle actin; OT, Oxythiamine; VIM, Vimentin

To assess the effect of TKT inhibition on cardiac fibrosis, western blotting, Sirius red, Masson and IHC staining were performed. Results demonstrated that compared with sham group, the degree of cardiac fibrosis in AMI mice significantly increased and fibrotic proteins expression were elevated, such as Fibronectin, α-SMA, VIM and connective tissue growth factor (CTGF). After treatment with TKT inhibitor, the degree of cardiac fibrosis and the expression of fibrotic proteins were markedly suppressed (Fig. 4A-F), indicating that TKT makes effect on cardiac fibrosis of AMI mice.

### TKT inhibitor suppressed NRCFs phenotypic transformation

**Fig. 5:**
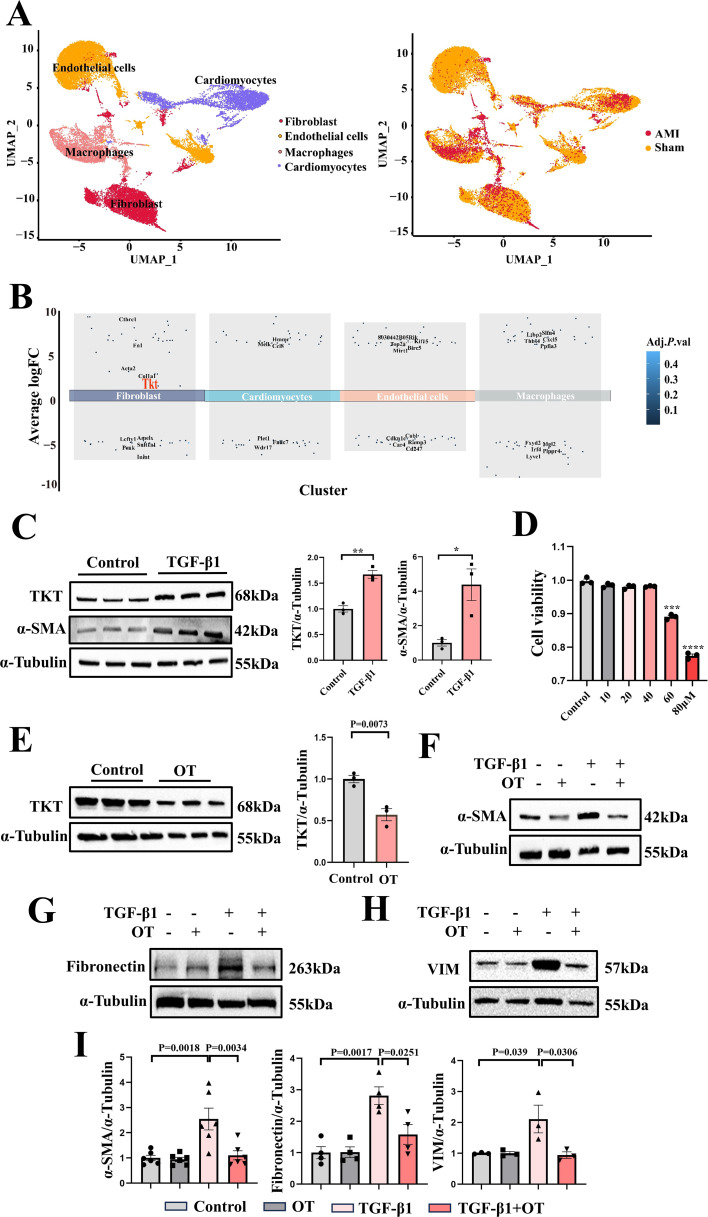
TKT was elevated in cardiac fibroblasts of AMI mice, and TKT inhibitor suppressed NRCFs phenotypic transformation. **A**: Bioinformatic analysis of GSE214611 for cardiac tissue in AMI mice. **B**: Bioinformatic analysis of GSE214611 for TKT upregulation in cardiac fibroblasts of AMI mice. **C**: Western blotting of TKT and α-SMA under TGF-β1 stimulation (*n* = 3). **D**: CCK-8 assay was used to explore the optimal concentration of OT on NRCFs activity. **E**: Western blotting of TKT inhibitor making effect (*n* = 3). **F-I**: Western blotting of α-SMA, Fibronectin and VIM expression under OT treatment (*n* = 3–6). Data were expressed as mean ± SEM. Two-tailed Unpaired t test was used to test the difference (**C-E**). One-way ANOVA test was used to test the difference (**I**). *P < 0.05, **P < 0.01, ***P < 0.001, **** *P* < 0.0001 vs. Control group (**C**, **D**). α-SMA indicates α-smooth muscle actin; OT, Oxythiamine; TGF-β1, transforming growth factor-β1; VIM, Vimentin

Analysis of dataset regarding cardiac tissue in AMI mice revealed that TKT was elevated in cardiac fibroblasts (Fig. 5A and B). To establish cardiac fibrosis cell model, NRCFs were incubated with 10 ng/ml TGF-β1 for 24 h. Both TKT and fibrotic protein such as α-SMA were significantly upregulated under TGF-β1 stimulation (Fig. 5C). To investigate the effect of TKT on NRCFs phenotypic transformation induced by TGF-β1, the optimal concentration of OT was determined to be 40 µM, which was based on previous studies [30–32] and CCK8 result (Fig. 5D), and the efficacy of OT was validated (Fig. 5E). NRCFs were pretreated with 40 µM OT for 2 h and then incubated with 10 ng/ml TGF-β1. We used α-SMA, Fibronectin and VIM to evaluate NRCFs phenotypic transformation, and found that OT pretreating significantly reduced TGF-β1-induced α-SMA, Fibronectin and VIM expression, indicating NRCFs phenotypic transformation was suppressed by TKT inhibition (Fig. 5F-I).

### TKT inhibitor suppressed proliferation and migration functions of NRCFs, and AKT phosphorylation

**Fig. 6:**
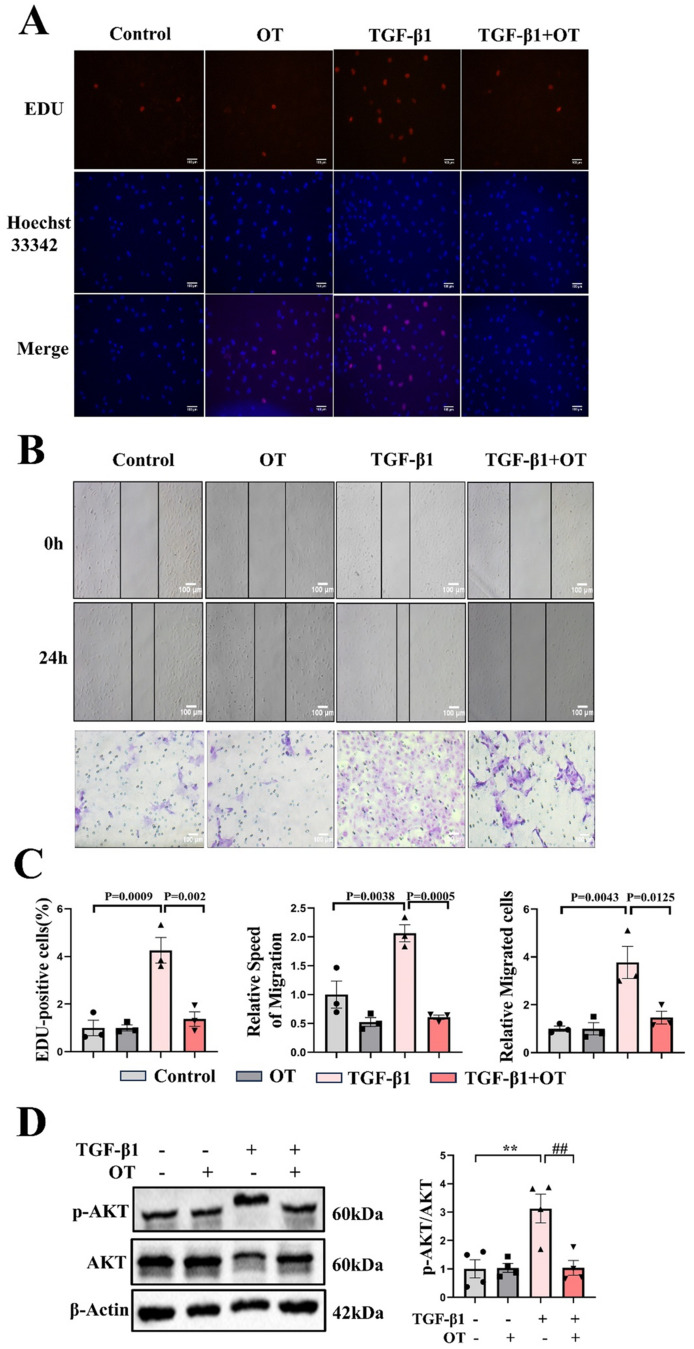
TKT inhibitor OT suppressed proliferation and migration of NRCFs, and AKT phosphorylation. **A**: EDU assay of NRCFs proliferation function upon TKT inhibition. **B**: Wound healing and Transwell assays of NRCFs migration function upon TKT inhibition. **C**: Quantitative tables for EDU proliferation, wound healing, and Transwell assays (*n* = 3). Scale bar: 100 μm. **D**: Western blotting of p-AKT upon TKT inhibition (*n* = 4). Data were expressed as mean ± SEM. One-way ANOVA test (**C**, **D**) was used to test the difference. **P < 0.01 vs. Control. ##*P* < 0.01 vs. TGF-β1 (**D**). OT indicates Oxythiamine; TGF-β1, transforming growth factor-β1

After treatment of NRCFs with TKT inhibitor OT, experiments including EDU, wound healing and Transwell assays demonstrated significant suppression of proliferation and migration functions in NRCFs (Fig. 6A-C). Our review of relevant literatures indicating that AKT activation plays a crucial role in cardiac fibrosis [24]. Furthermore, TKT involved in the pathological processes of colorectal and gastric cancer by activating AKT [18, 25]. Therefore, we proposed that the role of TKT in cardiac fibrosis post AMI was closely related to AKT activation. Western blotting result revealed that under TKT inhibition, AKT phosphorylation was suppressed, indicating that TKT attenuated cardiac fibrosis mainly by deactivating AKT pathway.

Following transfection with TKT overexpression (OE) plasmid, Western blotting confirmed successful OE of TKT (Figure S5A and E), accompanied by α-SMA upregulation (Figure S5B and F). Upon inhibition of AKT phosphorylation using 10 µM LY294002 following previous study [33], Western blotting demonstrated that α-SMA expression was not elevated even in the context of TKT OE (Figure S5C and D). Moreover, immunofluorescence assay demonstrated that the fluorescence levels of Fibronectin, α-SMA, and VIM were not increased upon TKT OE (Figure S5H and I), further indicating that TKT regulated NRCFs phenotypic transformation by p-AKT. Meanwhile, EDU proliferation, wound healing and Transwell assays demonstrated that NRCFs proliferation and migration were not enhanced by TKT OE (Figure S6A-C), further indicating that the pro-fibrotic effect of TKT on NRCFs was mediated by promoting AKT phosphorylation.

## Discussion

Previous studies have investigated TKT in conditions such as neoplastic diseases, diabetic cardiomyopathy, and unstable atherosclerotic plaque [16, 31, 34, 35]. However, no research has yet been conducted on TKT in relation to cardiac fibrosis following AMI. In this study, we firstly identified TKT was significantly elevated in serum and PBMCs of AMI patients. Analysis of AMI patients with left ventricular remodelling revealed that TKT level was closely associated with reduced cardiac function. Our clinical study also demonstrated that TKT level was markedly elevated in ACS patients and the clinical value of TKT in distinguishing AMI and UA patients. Subsequently, follow-up of MACE in ACS patients and Cox regression analysis confirmed that TKT may be implicated in prognostic prediction for ACS patients. The prognostic prediction model including TKT, constructed based on Cox proportional hazards regression model, also demonstrated excellent predictive value. Our study confirmed that TKT, either alone or in combination with other clinical risk factors, provides valuable clinical utility for both diagnosis and prognosis. Findings regarding other clinical variables consistent with previous studies [36, 37].

Through bioinformatic analysis and experimental validation, we confirmed that TKT was significantly elevated in AMI mice. Analysis of single-cell spatial transcriptomic dataset revealed that TKT was primarily distributed in the fibrotic region of the myocardium following AMI, indicating a close association between TKT and myocardial fibrosis in AMI. Treatment of AMI mice with TKT inhibitor resulted in markedly improved cardiac function and reduced cardiac fibrosis. These findings collectively demonstrated that TKT may play a role in cardiac fibrosis following AMI.

Our study has confirmed that TKT was significantly elevated in both AMI patients and animal models. Myocardial fibrosis is a characteristic pathological feature occurring in various chronic and acute cardiovascular diseases, including AMI [33, 38–40]. Following AMI, cardiac fibroblasts undergo phenotypic transformation from quiescent state to activated state, that is to say, cardiac fibroblasts transforming into myofibroblasts termed phenotypic transformation [41]. Myofibroblasts play a crucial role in promoting the progression of myocardial fibrosis [42, 43]. Therefore, we focused cellular experiments on NRCFs, with particular emphasis on the pathological process of NRCFs phenotypic transformation. Myofibroblasts are characterized by high expression of α-SMA, which also synthesize substantial amounts of ECM protein such as fibronectin [44, 45].

To further investigate the mechanism of TKT on cardiac fibrosis, we conducted experiments using NRCFs. TKT inhibition of NRCFs suppressed the expression of fibrotic proteins such as Fibronectin, α-SMA, and VIM, which was consistent with the fibrotic markers reported in previous study [46]. To further validate the interaction between TKT and p-AKT, NRCFs were treated with TKT OE plasmid and p-AKT inhibitor. Results confirmed that under TGF-β1 stimulation, TKT OE promoted phenotypic transformation, proliferation and migration of NRCFs, which could be reversed by inhibiting AKT phosphorylation. Han B’s research also confirmed that AKT was involved in cardiac fibrosis following AMI, consistent with our findings [47]. In addition, our study has several limitations. First, TKT is expressed in various organisms. OT acts as a specific inhibitor of TKT, although it was aimed to target cardiac TKT, due to it lacks tissue specificity, high-dose OT administered in vivo may affect TKT activity and metabolic homeostasis in other tissues, which could further lead to systemic metabolic alterations such as changes in glucose metabolism and nucleotide synthesis. Therefore, the observed phenomenon cannot be solely attributed to TKT inhibition in the heart, and potential systemic influences should be taken into consideration. Future animal experiments using cardiac fibroblast-specific TKT knockout mice will yield more robust and persuasive findings. Second, based on single-center clinical data, we constructed a clinical prediction model including TKT. Although it has been demonstrated that our clinical model has no multicollinearity, results obtained from multicenter clinical data will be more persuasive. Third, OT alleviated cardiac fibrosis of AMI mice, however, whether OT can be translated into clinical application requires clinical trials.

## Conclusion

In summary, we identified TKT as a potential regulator of cardiac fibrosis following AMI. Mechanistically, we demonstrated that TKT promoted cardiac fibrosis post AMI by modulating p-AKT signaling pathway. Our study provides TKT as a promising target for treating cardiac fibrosis after AMI.

## Supplementary Information

Below is the link to the electronic supplementary material.

Supplementary Material 1

## Data Availability

The original results in our study are presented in the article or supplementary material, further inquiries can contact the corresponding author via email.
