# Supplementary material for: TKT inhibition attenuates cardiac fibrosis in myocardial infarction through deactivating AKT signaling pathway
Source: J Transl Med. 2026 Apr 2;24:519. doi: 10.1186/s12967-026-08065-6 (PMC13088558; doi:10.1186/s12967-026-08065-6)
Supplement: Supplementary file 1 — Supplementary Material 1 [file 12967_2026_8065_MOESM1_ESM.docx]

**TKT inhibition attenuates cardiac fibrosis in myocardial infarction through** **deactivating AKT signaling pathway**

A total of 267 ACS patients were included in this study. TKT mRNA was significantly upregulated in serum of acute coronary syndrome (ACS) patients (GSE61145). Concurrently, our ELISA result demonstrated that TKT was elevated in serum of ACS patients (**Figure S1A**). The ACS patients were composed of 184 acute myocardial infarction (AMI) patients and 83 unstable angina (UA) patients, we found that TKT was significantly higher in AMI patients than UA patients (**Figure S1B and C**). Various parameters were compared between the AMI and UA group, and the differences were statistically significant. Detailed results are provided in **Table S1**.

Binary logistic regression analysis revealed significant differences in TKT (OR 1.409, 95% CI 1.031-1.925, P=0.032), Male (OR 4.544, 95% CI 2.135-9.671, P=0.000), Dyslipidemia (OR 5.814, 95% CI 3.058-11.11, P=0.000), WBC(OR 1.247, 95% CI 1.119-1.390, P=0.000), indicating that they were risk factors for AMI. Detailed results are presented in **Table S2**.

The nomogram model was constructed on the basis of the logistic regression analysis results between patients with AMI and the control group, including TKT, Male, WBC, Dyslipidemia (**Figure S2A**). The calibration curve expressed high predictive accuracy, with actual AMI risk closely aligned with predicted risk (**Figure S2B**). The ROC curve of the nomogram model was shown in **Figure S2C** (AUC=0.839, 95%CI:0.786-0.891; Bootstrap AUC=0.839,95% CI: 0.789-0.884), indicating that the nomogram model could be used to distinguish patients with AMI from UA controls. The decision curve analysis indicated the high net benefit of the nomogram model (**Figure S2D**). These results suggested the nomogram model has preliminarily potential value for clinical application.

To assess whether TKT has predictive value for ACS patients, we followed up major adverse cardiovascular events (MACE) including cardiac death, nonfatal myocardial infarction, readmission for unstable angina and heart failure during a median follow-up of 352 days, and MACE occurred in 59 ACS patients. We performed Cox regression analysis, and found that TKT, left ventricular ejection fraction (LVEF), fractional shortening (FS), Monocyte, Lymphocyte, Hematocrit, mean corpuscular hemoglobin concentration (MCHC), total cholesterol (TC), and low-density lipoprotein cholesterol (LDL-C) were statistically significant in univariate Cox regression analysis, and in multivariate Cox regression analysis, TKT (HR 1.24, 95% CI 1.04-1.48, P=0.018), Lymphocyte (HR 0.69, 95% CI 0.48-0.98, P=0.037), Hematocrit (HR 1.03, 95% CI 1.01-1.06, P=0.044), MCHC (HR 0.94, 95% CI 0.91-0.99, P=0.042), and LDL-C (HR 1.03, 95% CI 1.01-1.05, P=0.005) were statistically significant. Detailed results are presented in **Table S3**.

We constructed prognostic prediction model based on the results of Cox regression analysis, including TKT, Lymphocyte, Hematocrit, MCHC and LDL-C (**Figure S3A**). The calibration curve expressed high predictive accuracy, with actual MACE risk closely approaching predicted risk (**Figure S3B**). The ROC curve of the nomogram model was shown in **Figure S3C** (AUC=0.830, 95%CI:0.755-0.905), indicating the prognostic model can preliminarily predict whether MACE occur after ACS patients discharge. The decision curve analysis also expressed high net benefit of the prognostic model (**Figure S3D**). These results suggested that the prognostic model including TKT of preliminary value for predicting MACE of ACS patients.

We also provided the results of Kaplan-Meier survival analysis, **Figure S3E** showing that high TKT group (TKT≥2.289ng/ml) was associated with higher risk of MACE compared to low TKT group in patients with ACS (log-rank p <0.0001). These results further suggested that TKT has preliminary value for predicting MACE of ACS patients.


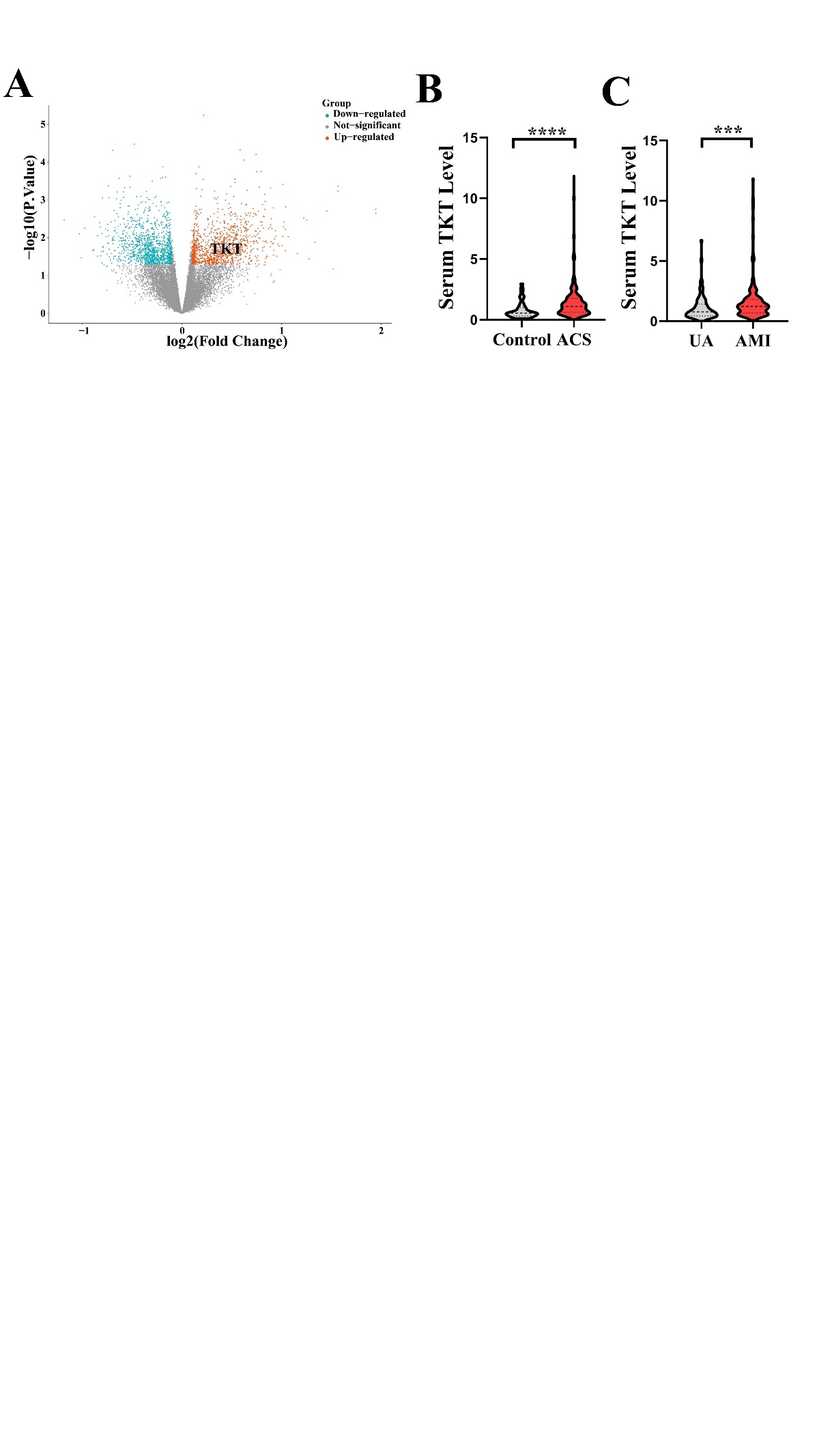


**Figure S1. TKT level in ACS patients.** A: Bioinformatic analysis of GEO RNA-seq data (GSE61145) for TKT level in serum of ACS patients. B: ELISA assay of TKT level in serum of ACS patients. C: ELISA assay of TKT level in serum of UA and AMI patients. ****P* < 0.001 vs. UA group；*****P* < 0.0001 vs. Control group. Mann-Whitney U test (B, C) was used to test the difference. ACS indicates acute coronary syndrome; AMI, acute myocardial infarction; UA, unstable angina.

**Table S1. The basic characteristics of AMI patients and control group.**

| **Characteristics** | **UA (n=83)** | **AMI (n=184)** | ***P* Value** |
| --- | --- | --- | --- |
| Age, years | 77.00[69.00,84.50] | 65.00[53.00,74.00] | <0.001 |
| Male, n(%) | 45(54.22) | 152(82.61) | <0.001 |
| Cigarette smoking, n(%) | 21(25.30) | 92(50.00) | <0.001 |
| Hypertension, n(%) | 62(74.70) | 130(70.65) | 0.593 |
| Dyslipidemia, n(%) | 25(30.12) | 129(70.11) | <0.001 |
| WBC, 10^9^ /L | 6.47[5.30,8.25] | 9.11[7.36,11.40] | <0.001 |
| Neutrophil, 10^9^ /L | 4.59[3.54,6.27] | 6.70[4.99,8.70] | <0.001 |
| TC, mmol/L | 3.63[2.91,4.54] | 4.56[3.73,5.40] | <0.001 |
| LDL-C, mmol/L | 2.21[1.66,2.74] | 2.70[2.15,3.36] | <0.001 |
| LDH(U/L) | 215[182.15,246.8] | 262[210.75,340] | <0.001 |
| Scr, umol/L | 79[63.70,100.85] | 73.10[58.50,87.60] | 0.049 |
| UA, umol/L | 357.2[258.45,425.8] | 345.45[290.05,411.7] | 0.003 |
| TKT(ng/mL) | 0.77[0.43,1.38] | 1.22[0.68,1.80] | <0.001 |
| Nitrate, n(%) | 58(69.88) | 183(99.46) | <0.001 |
| Antiplatelet, n(%) | 61(73.49) | 182(98.91) | <0.001 |
| β-blockers, n(%) | 57(68.67) | 160(86.96) | 0.001 |
| Statin, n(%) | 70(84.34) | 182(98.91) | <0.001ZHU |

LDH, lactate dehydrogenase; LDL-C, low-density lipoprotein cholesterol; Scr, serum creatinine; TC, total cholesterol; UA, uric acid; WBC, white blood cell.

**Table S2. Binary logistic regression analysis of** **risk factors in AMI patients.**

| **Characteristics** | **OR** | **95%CI** | ***P* Value** |
| --- | --- | --- | --- |
| Cigarette smoking | 1.435 | 0.700-2.941 | 0.323 |
| Male | 4.544 | 2.135-9.671 | 0.000 |
| TKT | 1.409 | 1.031-1.925 | 0.032 |
| WBC | 1.247 | 1.119-1.390 | 0.000 |
| Dyslipidemia | 5.814 | 3.058-11.11 | 0.000 |

WBC, white blood cell.


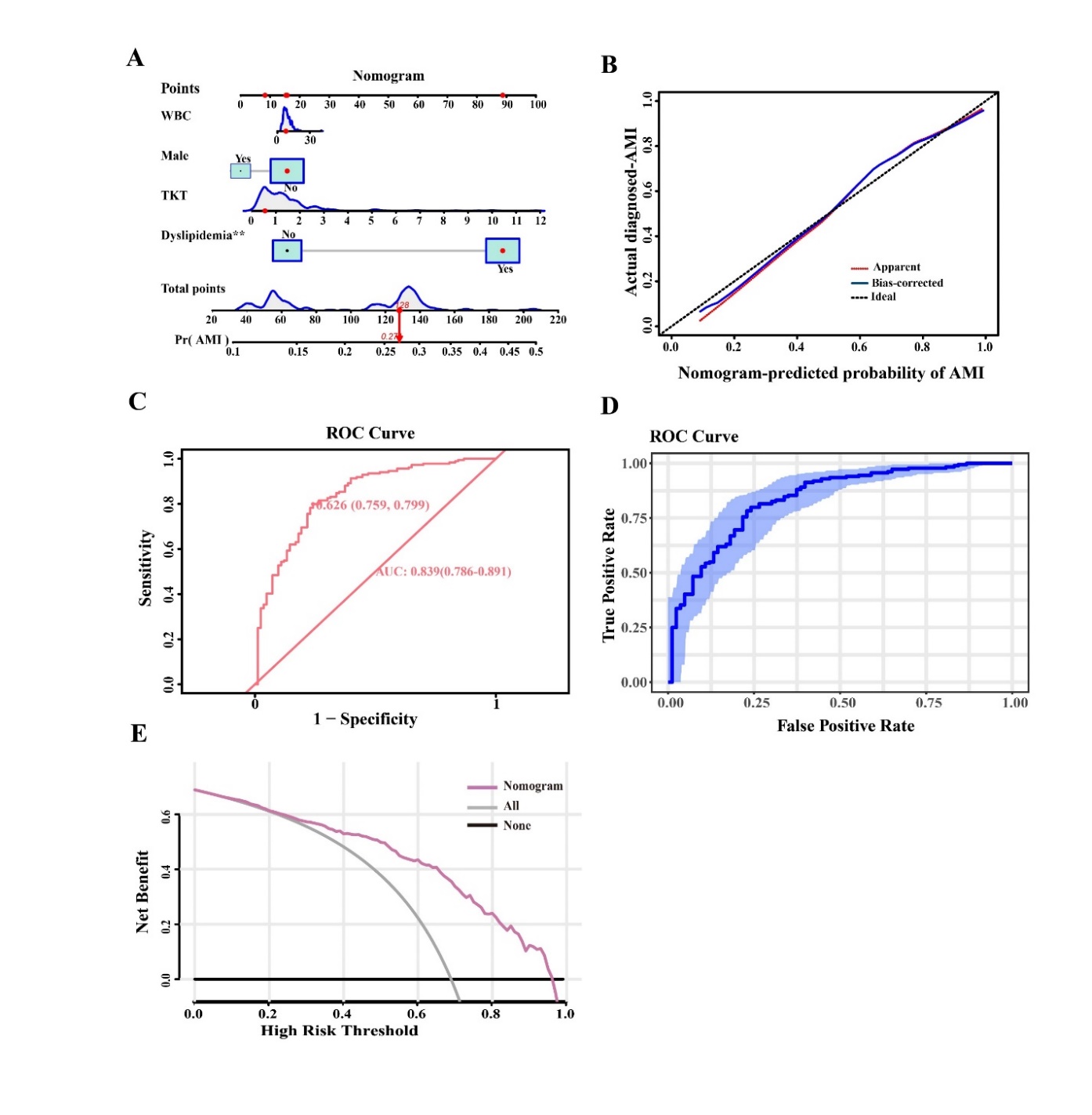


**Figure S2. Construction and evaluation of the clinical prediction model of AMI.** A: Nomogram-based clinical prediction model using independent risk factors in AMI patients. B: Calibration curve assessing the predictive accuracy of the Nomogram model. C: ROC curve of the Nomogram model. D: ROC curve using Bootstrap internal validation (resampling = 500). E: DCA curve evaluating the clinical net benefit of the Nomogram model. AMI, acute myocardial infarction; WBC indicates white blood cell.

**Table S3. Cox regression analysis of MACE in ACS patients.**

| Characteristics | HR | 95％Cl | P | HR | 95％Cl | P |
| --- | --- | --- | --- | --- | --- | --- |
| Age, years | 0.98 | 0.97-1 | 0.057 |  |  |  |
| Male, n(%) | 0.95 | 0.51-1.76 | 0.86 |  |  |  |
| Cigarette smoking, n(%) | 1.62 | 0.97-2.7 | 0.067 |  |  |  |
| Hypertension, n(%) | 0.74 | 0.42-1.33 | 0.316 |  |  |  |
| Dyslipidemia, n(%) | 1.67 | 0.92-3.04 | 0.093 |  |  |  |
| LVESD(mm) | 0.97 | 0.93-1.02 | 0.276 |  |  |  |
| LVEDD(mm) | 1.01 | 0.97-1.05 | 0.502 |  |  |  |
| LVEF(%) | 1.06 | 1.03-1.1 | 0.001 | 1.22 | 1-1.5 | 0.055 |
| LVFS(%) | 1.08 | 1.03-1.13 | 0.001 | 0.83 | 0.64-1.08 | 0.174 |
| WBC, 10^9^ /L | 0.97 | 0.9-1.05 | 0.46 |  |  |  |
| Neutrophil, 10^9^ /L | 1.00 | 0.94-1.07 | 0.91 |  |  |  |
| Monocyte, 10^9^ /L | 0.29 | 0.09-0.96 | 0.043 | 0.36 | 0.09-1.41 | 0.143 |
| Lymphocyte, 10^9^ /L | 0.69 | 0.5-0.94 | 0.021 | 0.69 | 0.48-0.98 | 0.037 |
| RBC, 10^9^ /L | 1.14 | 0.8-1.62 | 0.473 |  |  |  |
| Hematocrit (%) | 1.04 | 1.01-1.06 | 0.003 | 1.03 | 1.01-1.06 | 0.044 |
| MCHC, g/L | 0.95 | 0.92-0.98 | 0.028 | 0.94 | 0.91-0.99 | 0.042 |
| TC, mmol/L | 1.01 | 1.001-1.02 | 0.025 |  |  |  |
| TG, mmol/L | 1.05 | 0.94-1.17 | 0.418 |  |  |  |
| LDL-C, mmol/L | 1.02 | 1.01-1.04 | 0.019 | 1.03 | 1.01-1.05 | 0.005 |
| TKT(ng/mL) | 1.36 | 1.17-1.58 | 0.00 | 1.24 | 1.04-1.48 | 0.018 |
| Nitrate, n(%) | 2.89 | 0.7-12 | 0.144 |  |  |  |
| Antiplatelet, n(%) | 4.65 | 0.64-34.03 | 0.13 |  |  |  |

ACS, acute coronary syndrome; LDL-C, low-density lipoprotein cholesterol; LVESD，Left ventricular end systolic diameter; LVEDD，Left ventricular end diastolic diameter; LVEF, Left ventricular ejection fraction; LVFS, Left ventricular fractional shortening; MACE, major adverse cardiovascular events; MCHC，mean corpuscular hemoglobin concentration; RBC，red blood cell；TC, total cholesterol; TG，triglycerides; WBC, white blood cell.


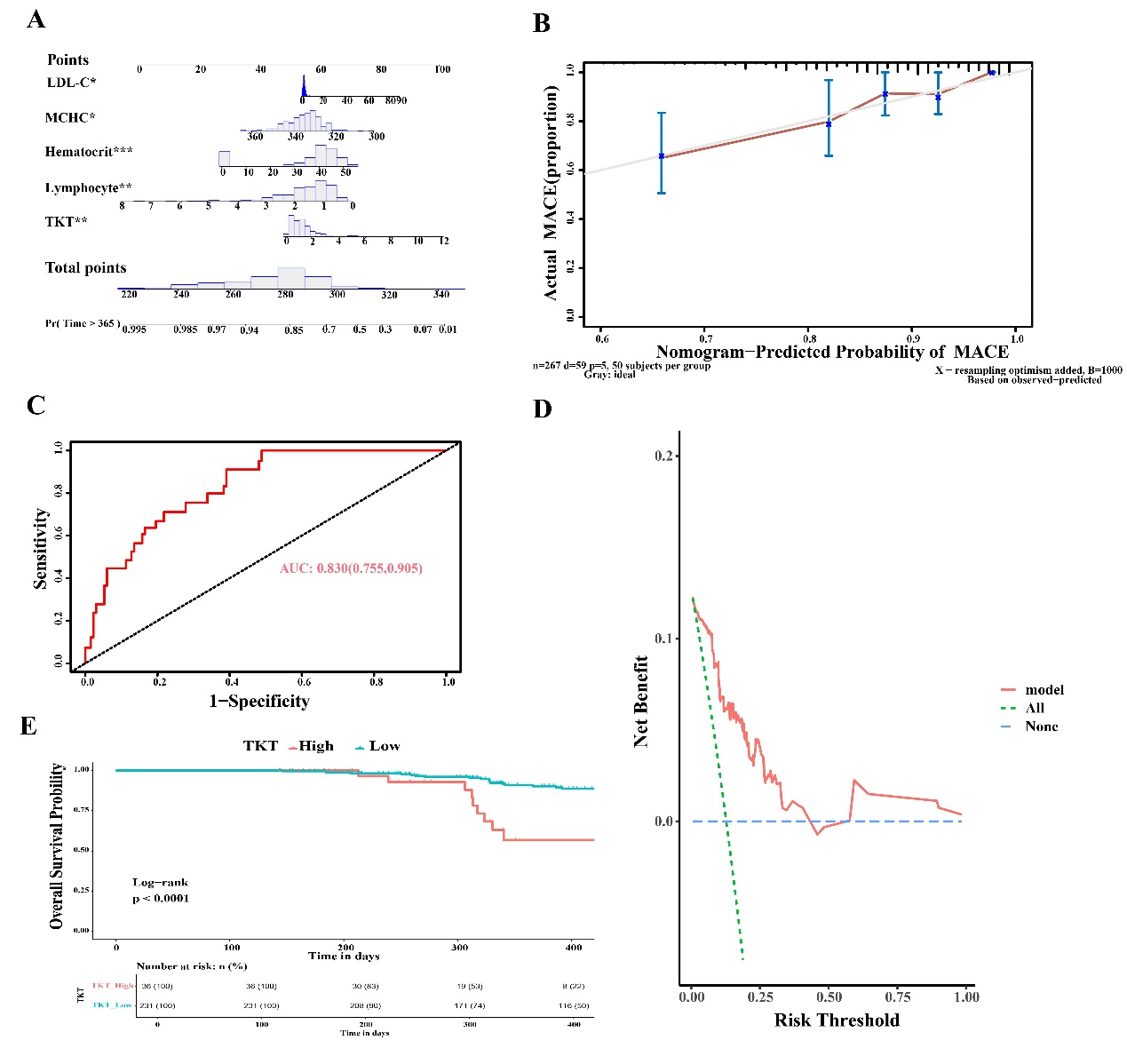


**Figure S3. Development of a prognostic prediction model of MACE in ACS patients.** A: Prognostic prediction model of MACE in ACS patients. B: Calibration curve assessing the predictive accuracy of the prognostic model. C: ROC curve evaluating the diagnostic efficacy of the prognostic model. D: DCA curve evaluating the clinical net benefit of the prognostic model. E: Kaplan-Meier curves of high TKT and low TKT group. LDL-C indicates low-density lipoprotein cholesterol; MACE, major adverse cardiovascular events; MCHC, mean corpuscular hemoglobin concentration.


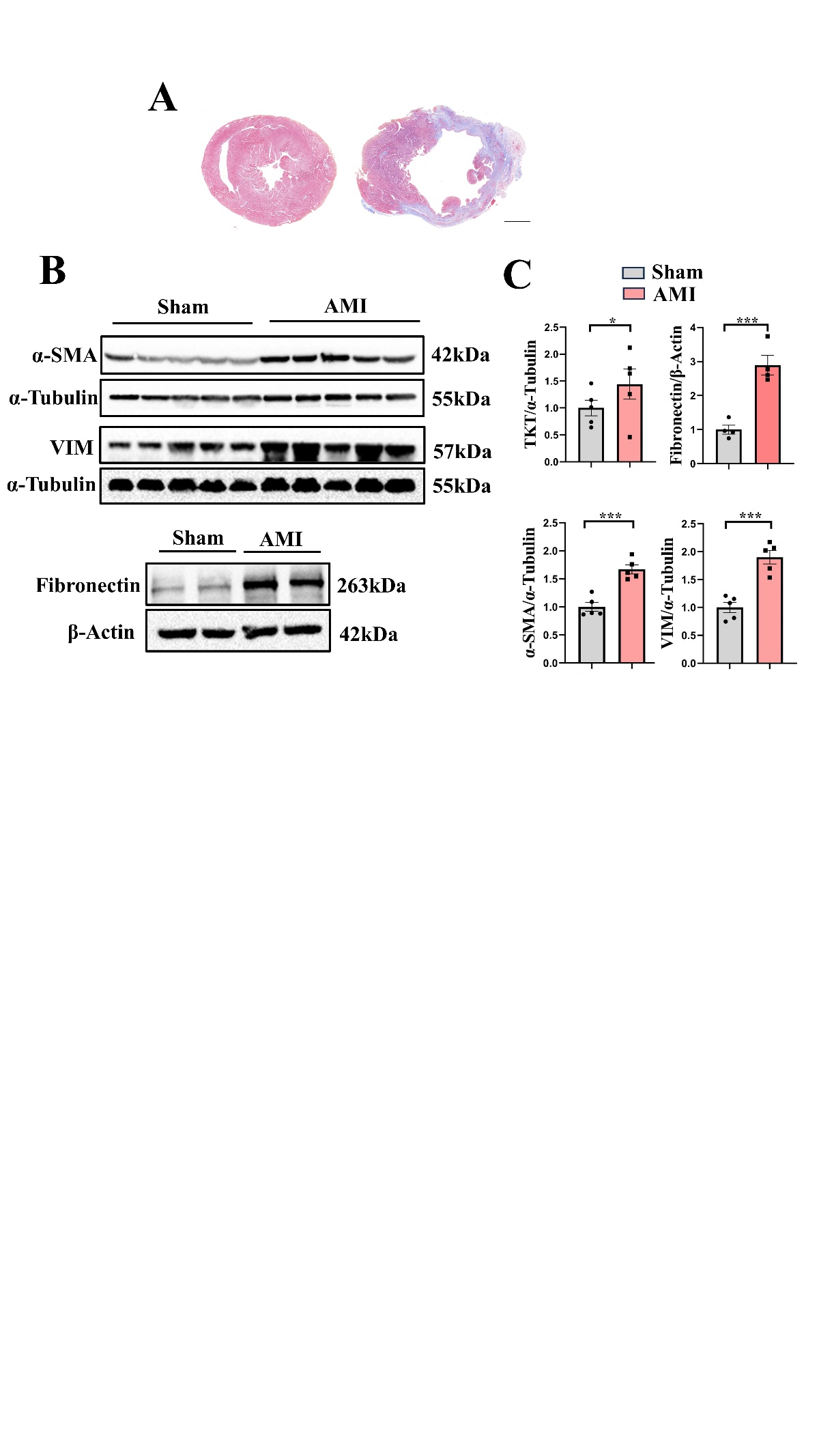


**Figure S4. TKT level in AMI model.** A: Masson staining confirmed successful establishment of AMI animal model. B-C: Western blotting of TKT and cardiac fibrosis proteins including Fibronectin, α-SMA, and VIM in AMI model (n=4-5). Two-tailed Unpaired t test (C) was used to test the difference, **P* < 0.05, ****P* < 0.001 vs. Sham. Scale bar 1000μm. AMI indicates acute myocardial infarction; α-SMA, α-smooth muscle actin; VIM, Vimentin.


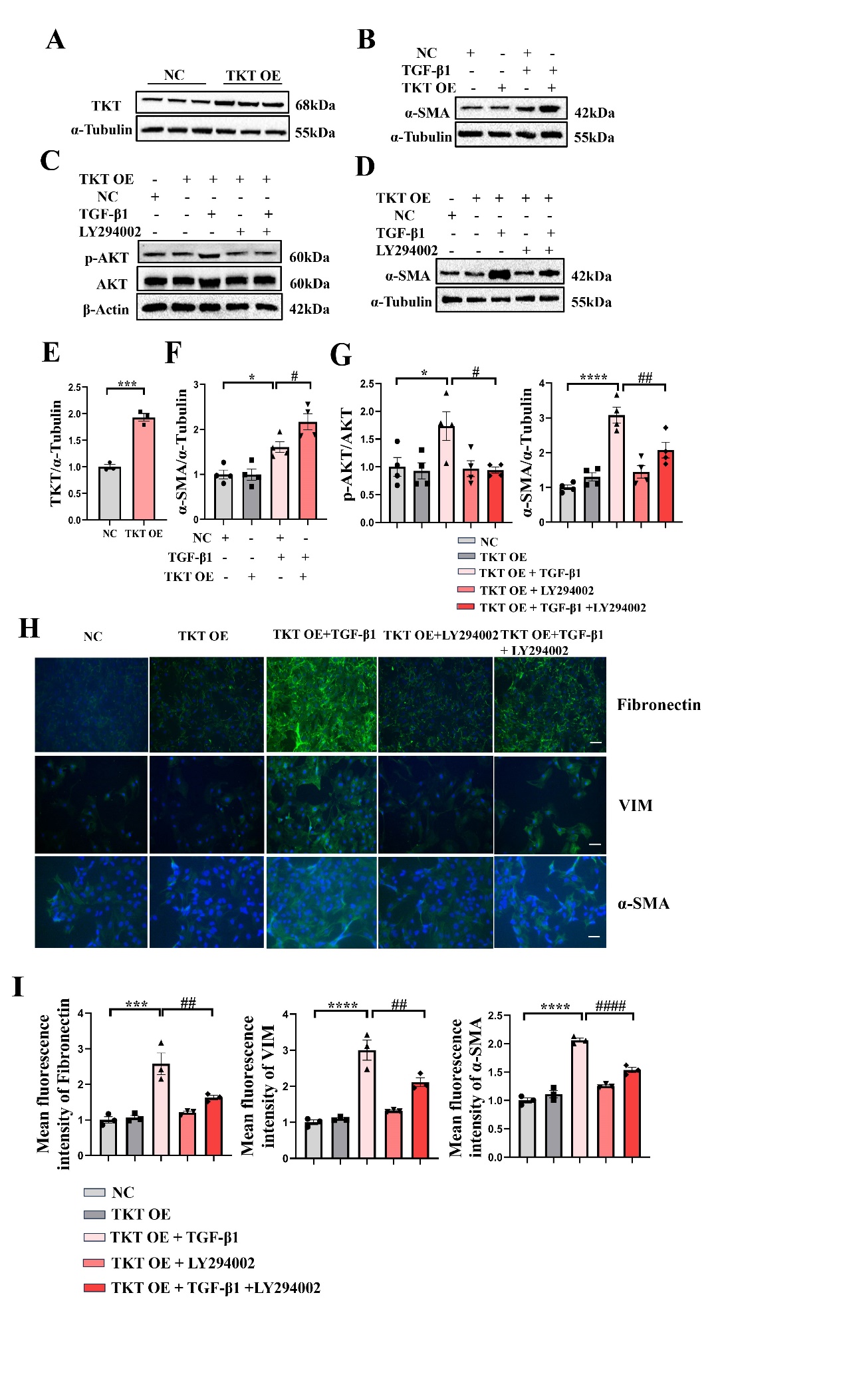


**Figure S5. TKT OE regulated NRCFs phenotypic transformation by promoting AKT phosphorylation.** A, E: Western blotting of TKT OE (n=3). B, F: Western blotting of α-SMA following TKT OE (n=4). C, D, G: Western blotting of p-AKT and α-SMA following TKT OE with p-AKT inhibitor LY294002 treatment (n=4). H, I: Immunofluorescence assay of Fibronectin, α-SMA, and VIM (n=3). Scale bar: 100μm. Two-tailed Unpaired t test was used to test the difference (E), One-way ANOVA test was used to test the difference (F, G, I). **P* < 0.05, ****P* < 0.001, *****P* < 0.0001 vs. NC group (E-G, I). #*P* < 0.05 vs. TGF-β1+NC group (F), TKT OE + TGF-β1 group (G), ##*P* < 0.01, ####*P* < 0.0001 vs. TKT OE+TGF-β1 group (G, I). α-SMA indicates α-smooth muscle actin; NC, negative control; OE, overexpression; TGF-β1, transforming growth factor-β1; VIM, Vimentin.


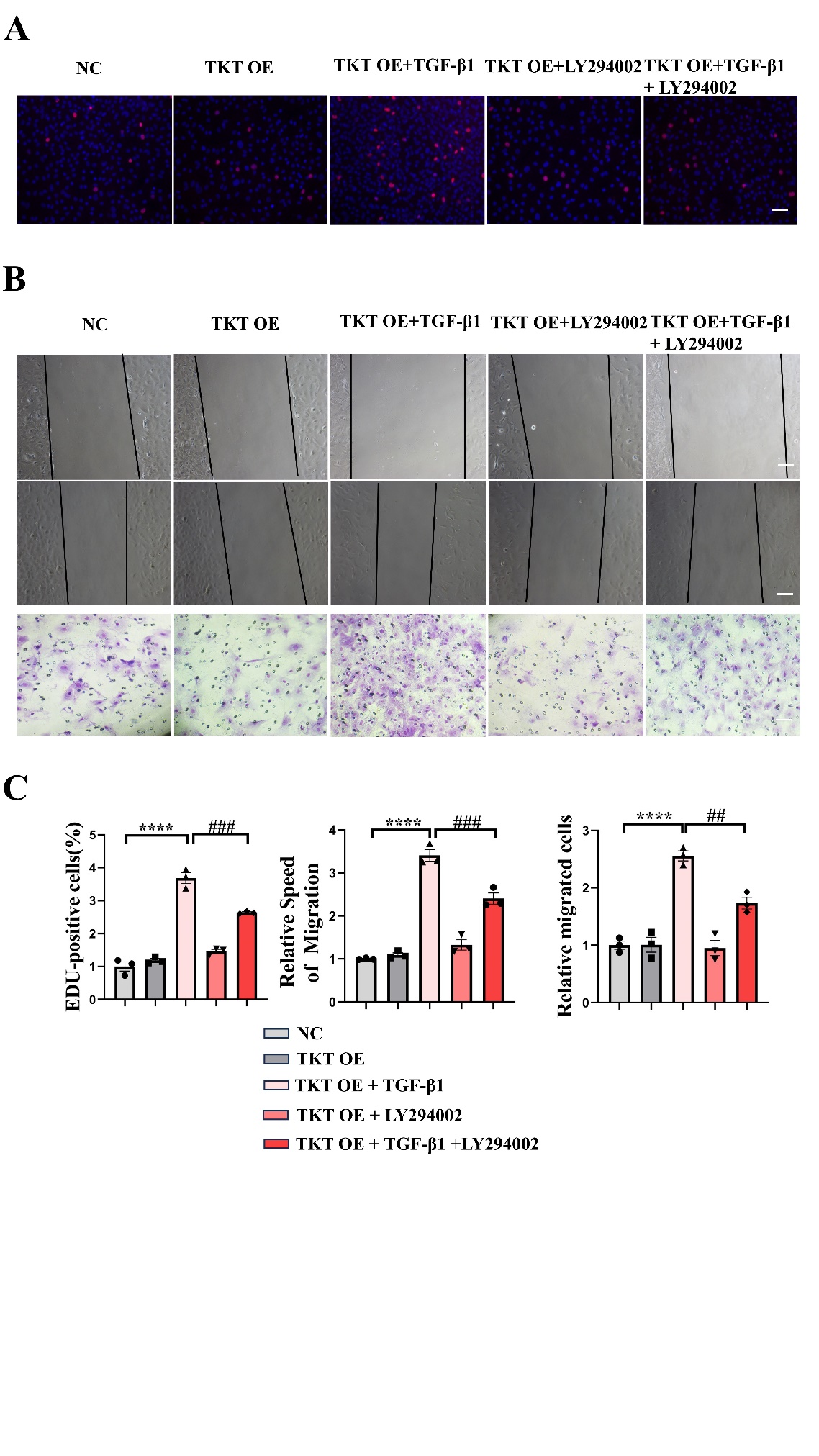


**Figure S6. TKT OE regulated NRCFs proliferation and migration by promoting AKT phosphorylation.** A-C: EDU proliferation, wound healing, and Transwell assays. n=3 per group. Scale bar: 100 μm. One-way ANOVA test was used to test the difference(C). *****P* < 0.0001 vs. NC group. ##*P* < 0.01, ###*P*< 0.001 vs. TKT OE+TGF-β1 group. NC, negative control; OE, overexpression; TGF-β1, transforming growth factor-β1.
